# GIS-aided planning of insecticide spraying to control dengue transmission

**DOI:** 10.1186/1476-072X-12-42

**Published:** 2013-09-25

**Authors:** Hone-Jay Chu, Ta-Chien Chan, Fang-Ju Jao

**Affiliations:** 1 Department of Geomatics, National Cheng Kung University, No. 1, University Road, 701 Tainan City, Taiwan; 2 Research Center for Humanities and Social Sciences, Academia Sinica, 128 Academia Road, Section 2, 115 Nankang, Taipei, Taiwan

**Keywords:** Integer programming, Dengue fever, Insecticide spraying planning, Epidemic risk, GIS, Multi-objective

## Abstract

**Background:**

The purpose of this paper is to integrate a multi-objective integer programming formulation and geographic information system (GIS) into dynamically planning the insecticide spraying area for preventing the transmission of dengue fever.

**Methods:**

The optimal spraying area to combat dengue infections is calculated by the multi-objective integer programming model using the dengue epidemic in 2007 in Tainan City of southern Taiwan and is compared with the areas actually sprayed by the local health department. The dynamic epidemic indicators (i.e. frequency, intensity and duration) that identify major temporal characteristics of the dynamic process of an epidemic are all incorporated into the model.

**Results:**

The results indicate that the model can design the spraying area effectively when the trade-off between the coverage of dengue epidemics risk and area compactness is considered.

**Conclusions:**

The model provides an alternative way to obtain a cost-effective spraying area in controlling future dengue epidemics. The proposed model in this study will be beneficial for strategically allocating dengue control resources.

## Introduction

Dengue fever is a high-incidence, vector-borne infectious disease, and an estimated 390 million (95% credible interval 284-528 million) dengue infections occur per year around the world, chiefly distributed in tropical countries [1]. Although a vaccine for dengue prevention is under development, there is still no good pharmaceutical intervention to prevent infection [2]. Therefore, public health efforts focus on environmental control and surveillance of the mosquitoes’ indexes, including the House Index (HI), Container Index (CI) and Breteau Index (BI) [3,4]. During the initial stage of an epidemic, local health departments will mobilize insecticide spraying resources within peridomestic space to prevent dengue vector breeding. The high costs associated with extensive spraying acknowledge that many residents may be inconvenienced. Based on one experimental study in southern Taiwan, the effectiveness of insecticide spraying within a radius of 100 meters can achieve a 90% reduction in the number of *Aedes aegypti* outdoors [5]. In traditional practice, the spraying area focused on the infected cases near large communities. However, this strategy is feasible only when there are few, sporadic infections in the initial stage of the epidemic. When numerous dengue infections occur in close temporal proximity, spraying areas will overlap and expand while limited human resources. It will be a big challenge for the frontline public health workers to control dengue transmission without a systematic control strategy. Therefore, we develop one mathematical model which considers multiple objectives with maximal coverage of dengue fever hotspots and a minimal boundary length of spraying area at the same time. In addition, the epidemic conditions including the disease’s frequency, intensity and duration are incorporated into the strategy behind the extent of spraying [6]. Geographic information systems (GIS) have provided useful tools for managing and surveillance of dengue fever [7,8]. This study integrates GIS and an integer programming formulation [9] to dynamically plan the insecticide spraying area for prevention of the transmission of dengue fever. An integer programming approach was developed to help us to identify the insecticide spraying area that maximizes coverage of epidemic risk spots and minimizes the length of the boundaries of sprayed areas. The epidemic conditions were built into our GIS planning model. The 2007 dengue epidemic in Tainan City, located in southern Taiwan, was utilized as a case study and to compare the difference between the GIS-aided strategy and the empirical spraying area.

## Methods

### Material and study area

**Figure 1:**
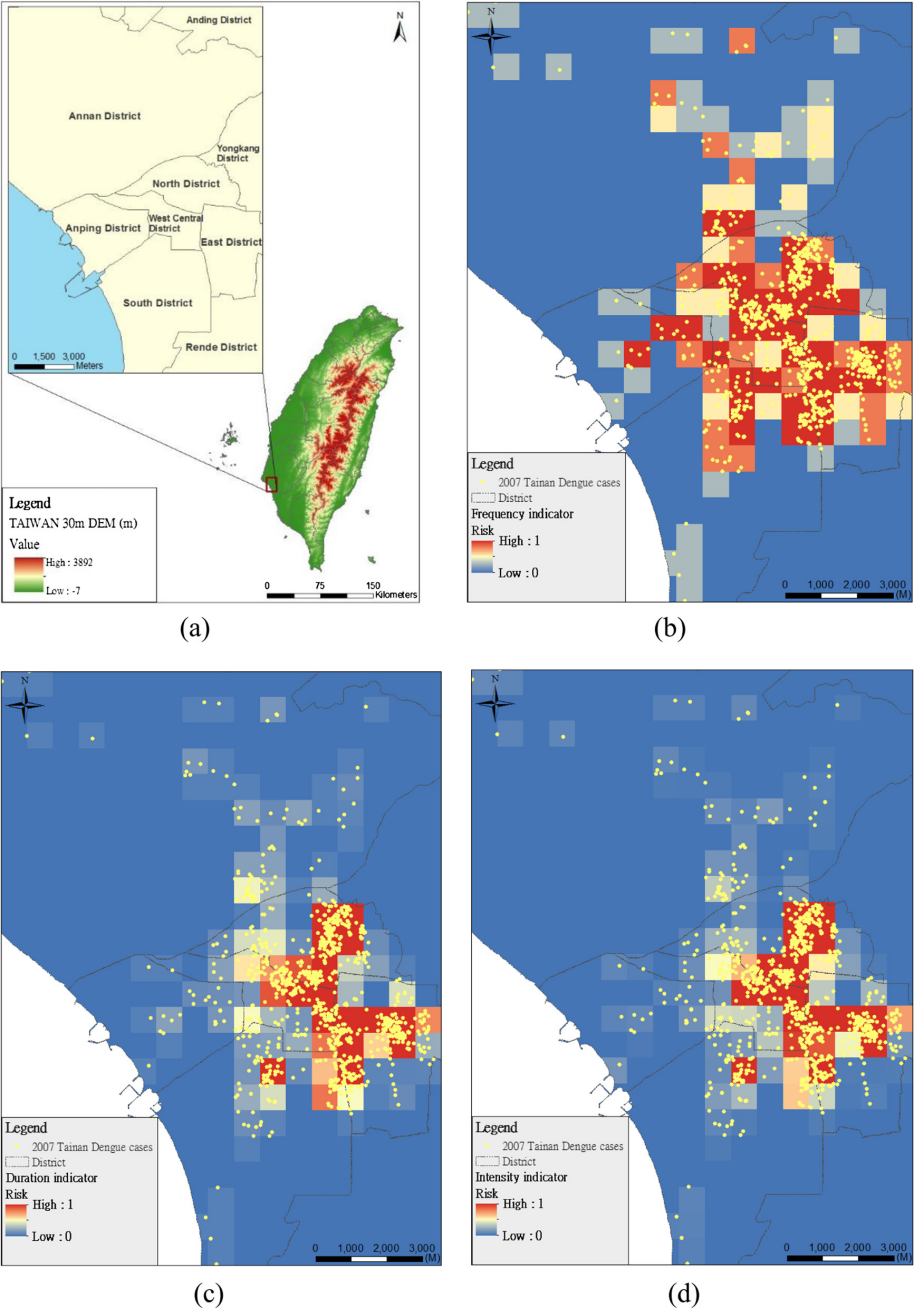
**Three indicators of epidemic risk: frequency, duration, and intensity. (a)** study area in south Taiwan; **(b)** frequency indicator of epidemic risk; **(c)** duration indicator of epidemic risk; **(d)** intensity indicator of epidemic risk.

The study area was in Tainan city, Taiwan (Figure 1a). It is the oldest city in Taiwan, and the second largest in southern Taiwan. Modern Tainan was founded under a program of urban redevelopment. The area is 175.65 square kilometers and the population was 764,658 in 2007. From June to December in 2007, Tainan city recorded a total of 1,197 cases of dengue infections, according to the national notifiable diseases surveillance system of Taiwan’s CDC (Table 1). The locations of the anonymous infection cases were geographically masked by the shifting distance method to preserve the clustering pattern and protect the cases’ privacy [10]. The insecticide spraying information including the spraying date and the spraying area was obtained from the Department of Health in Tainan City. The study was approved by the institutional review board (IRB) of Academia Sinica.

**Table 1:** The temporal distribution of dengue infections from Jun. 2007 to Dec. 2007

**Month**	**Jun.**	**Jul.**	**Aug.**	**Sep.**	**Oct.**	**Nov.**	**Dec.**	**Total**
Number of dengue infections	8	60	137	160	362	373	97	1,197

Integrating a multi-objective integer programming formulation using GIS in this study is to plan the optimal insecticide spraying area so as to maximize coverage of areas with dengue fever epidemic risk, minimize the length of the boundaries of sprayed areas, and take into account total area constraints. The epidemic risk goals can be related to (1) how often the disease occurs (frequency, α), (2) how long an epidemic persists (duration, β) and (3) how significant cases occurring in consecutive weeks are during an epidemic (intensity, γ). These three indicators, however, represent the disease component of dengue risk and enable to identify high-risk areas (hotspots) for the occurrence of dengue fever [6].

The planning model is described as:

(1) $$\mathrm{Max}\sum_{i=1}^{n}\left(w_{1}S_{i}I_{i}-w_{2}\sum_{j\in C_{i}}L_{\mathrm{ij}}\left(P_{\mathrm{ij}}+N_{\mathrm{ij}}\right)\right)$$

s.t.

(2) $$S_{i}=w_{\alpha }\alpha_{i}+w_{\beta }\beta_{i}+w_{\gamma }\gamma_{i}$$

(3) $$\sum_{i=1}^{n}I_{i}=z$$

(4) $$I_{i}-I_{j}-P_{\mathrm{ij}}+N_{\mathrm{ij}}=0\;\forall i,j\in C_{i}$$

(5) $$P_{\mathrm{ij}}+N_{\mathrm{ij}}\leq 1$$

Where *S*_*i*_ is epidemic risk in combination of frequency (α_*i*_), duration (β_*i*_) and intensity (γ_*i*_); *L*_*ij*_ is the length of the border between cells *i* and *j*; *z* is the planning area of the dengue hotspot zone; in this study, the cell size is 0.5 km^2^ (the average size of villages in Tainan city is 0.79 km^2^) and z is set as 15 or 25 km^2^. *I*_*i*_ is a binary number that represents whether cell *i* is a hotspot or not*; P*_*ij*_ and *N*_*ij*_ are pseudo-binary numbers that are related to the boundary determination. *P*_*ij*_ = 1 if *I*_*i*_ = 1 and *I*_*i*_ =0, *P*_*ij*_ = 0, otherwise; *N*_*ij*_ =1 if *I*_*j*_ = 1 and *I*_*i*_ =0, *N*_*ij*_ = 0, otherwise. *n* is the number of cells in the study area. *C*_*i*_ is the set of cells adjacent to cell *i*. *w*_1_ and *w*_2_ are the weights of epidemic risk coverage and boundary length. *w*_α_,*w*_β_ and *w*_γ_ are the weights of frequency, duration and intensity indicators. For details of the optimization model, please refer to the reference [9]. This model was programmed by LINGO (Lindo Systems, Inc., Chicago, IL, USA).

The frequency indicator at cell *i* (α_*i*_) of a disease during an epidemic can be defined by a probability that one or more laboratory-confirmed cases occurred in certain months out of the total epidemic period.

(6) $$\alpha_{i}=\frac{{\mathrm{EW}}_{i}}{{\mathrm{TW}}_{i}}$$

Where TW_*i*_ is the total number of months during the entire epidemic period. EW_*i*_ is the total number of months in which one or more cases occurred during the entire epidemic period.

The duration indicator at cell *i* (β_*i*_) can be used as the average number of months of an epidemic wave.

(7) $$\beta_{i}=\frac{{\mathrm{EW}}_{i}}{{\mathrm{TV}}_{i}}$$

where TV_i_ is the total number of epidemic waves during the entire epidemic period.

The intensity indicator at cell *i* (γ_*i*_) refers to the likely magnitude within an epidemic wave.

(8) $$\gamma_{i}=\frac{{\mathrm{IR}}_{i}}{{\mathrm{TV}}_{i}}$$

Where IR_*i*_ is the incidence rate during the defined epidemic period.

## Results

Figure 1 shows three epidemic indicators which are frequency (Figure 1b), duration (Figure 1c) and intensity (Figure 1d) in the study area, respectively. The indicators demonstrate major temporal characteristics and patterns of the epidemic dynamic process. The distribution of the frequency indicator is more widely diffused than the distribution of the other two indicators (i.e. duration and intensity). The high frequency indicators are located in the North, East and Middle-West Districts. The spatial patterns of duration and intensity indicators are consistent and highly correlated (correlation coefficient = 0.97, p-value <0.01), and were done by SPSS 10.0 (SPSS, Inc., Chicago IL). The model determines the insecticide spraying areas for reaching two objectives such as maximum coverage of environmental epidemic risk and minimizing the boundary of the areas. The compactness of the area is beneficial for environmental management and control.

**Figure 2:**
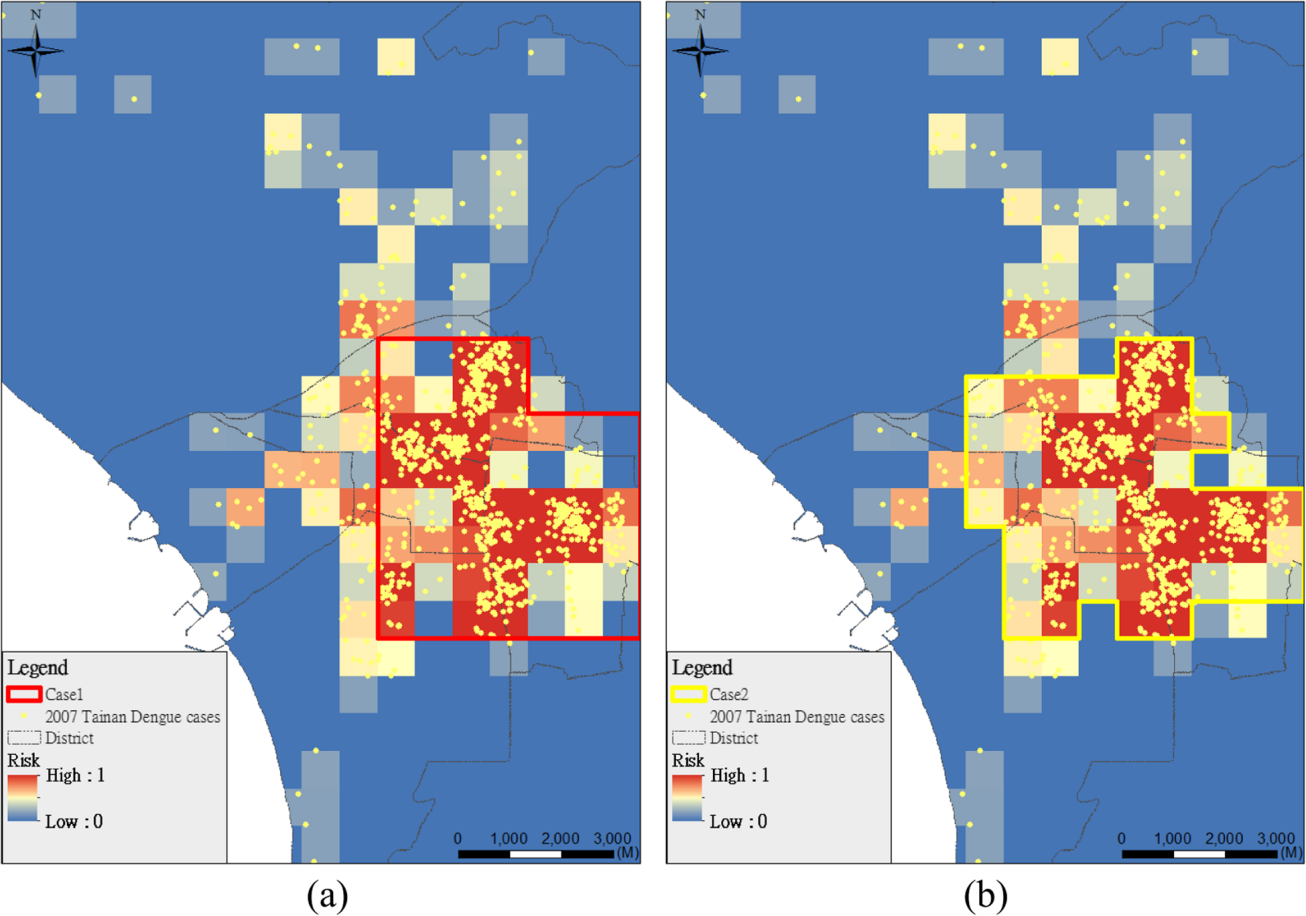
**Planning area based on different weights between maximal coverage of epidemic risk (*w***_**1**_**) and minimal boundary (*w***_**2**_**). (a)** Case 1 (*w*_1_:*w*_2_ = 1:1); **(b)** Case 2 (*w*_1_:*w*_2_ = 1:0.25).

**Figure 3:**
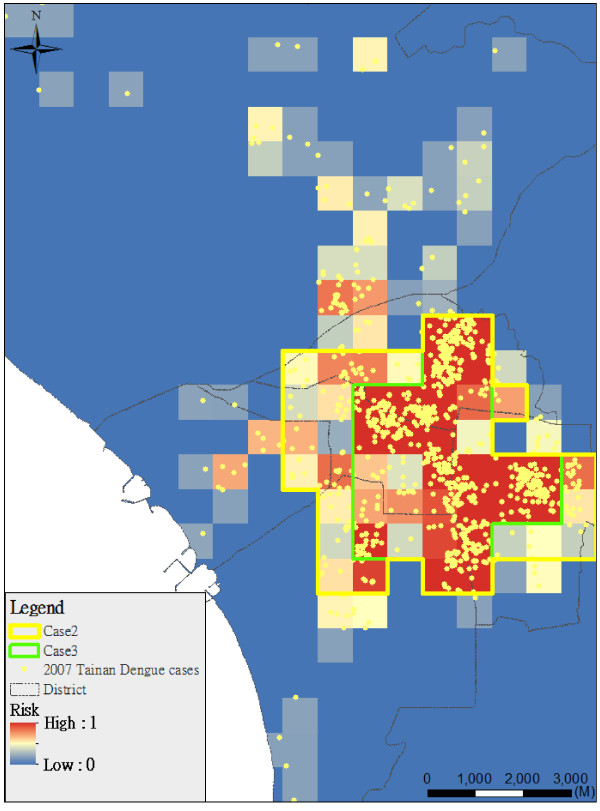
**Planning area comparisons based on different area constraints (Case 2: 25 km**^**2**^**; Case 3: 15 km**^**2**^**).**

Figure 2 shows the optimal insecticide spraying areas at different weights of boundary minimizing (Case 1, w_2_ = 1 (Figure 2a); Case 2, w_2_ = 0.25 (Figure 2b)). In the case with high weight (Case 1) of boundary minimization, the spraying areas are more compact, and the average epidemic risk in the condition is 0.28. However, the hotspot area is less compact and more effective at covering area of the higher epidemic risk (average is 0.31) if the model places a low weight on boundary minimization (Case 2). Figure 3 shows the planning cases considering different area sizes in the case study (Case 2: 25 km^2^; Case 3: 15 km^2^). The area requiring spraying will be adapted over time; in the initial stage of an epidemic, the requirement area may be large and then the requirement area decreases when the epidemic is under control.

**Figure 4:**
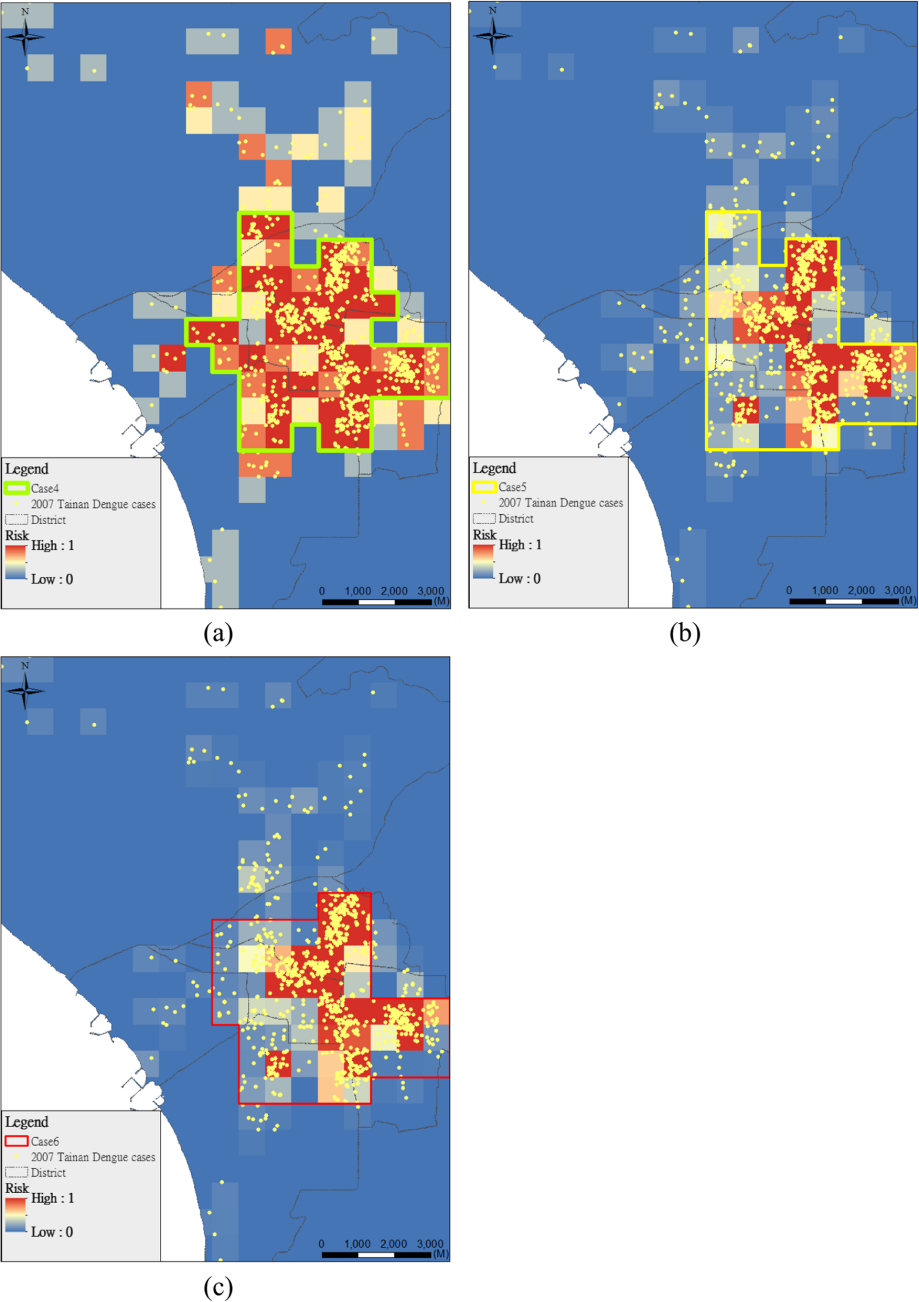
**Planning area considering different weights of indicators (Cases 4-6). (a)** Case 4 (*w*_α_:*w*_β_:*w*_γ_ = 2:1:1), **(b)** Case 5 (*w*_α_:*w*_β_:*w*_γ_ = 1:2:1), **(c)** Case 6 (*w*_α_:*w*_β_:*w*_γ_ = 1:1:2).

**Figure 5:**
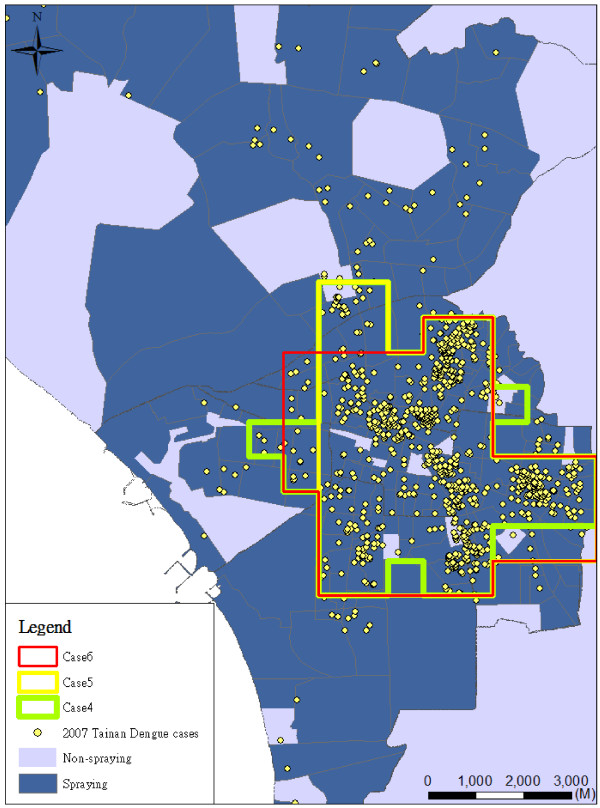
**Comparisons in Cases 4-6 with the spraying in real case.** The background in dark blue is the real insecticide spraying area for epidemic prevention in 2007.

Figure 4 shows the spraying area considering different combinations of epidemic risk indicators by different weights (i.e. Cases 4-6, 2:1:1 (Figure 4a), 1:2:1 (Figure 4b), and 1:1:2 (Figure 4c) in frequency, duration and intensity indicators). Most planning areas (84%) are consistent in Cases 4-6 (Figure 5). The results of spraying area are not significantly sensitive at different weights of epidemic risk indicators in Cases 4-6 (Table 2). Figure 5 also shows the spatial patterns of the planning area and real (historical) insecticide spraying area. The background in dark blue is the real insecticide spraying area for epidemic prevention in 2007. Our result shows that the area planned by our model is only 21% of the historical spraying area. In the spraying area, there are many possible mosquito breeding sites such as abandoned water towers, empty houses and open bare land.

**Table 2:** The combinations of the parameters in the case studies and the epidemic risk captured by the model

	**The parameters in each case**			**Average frequency indicator in planning area**	**Average duration indicator in planning area**	**Average intensity indicator in planning area**	**Average Epidemic risk**	**Boundary length (km)**
	***w***_**1**_**:*w***_**2**_	***w***_**α**_**:*w***_**β**_**:*w***_**γ**_	**Area (km**^**2**^**)**					
Case 1	1:1	1:1:1	25	0.491	0.166	0.188	0.282	21.2
Case 2	1:0.25	1:1:1	25	0.551	0.173	0.195	0.306	26.9
Case 3	1:0.25	1:1:1	15	0.633	0.256	0.297	0.395	19.8
Case 4	1:0.25	2:1:1	25	0*.*566	0.197	0.176	0.376	31.1
Case 5	1:0.25	1:2:1	25	0.545	0.195	0.175	0.271	25.5
Case 6	1:0.25	1:1:2	25	0.540	0.194	0.173	0.275	24.0

## Discussion

In this study, we demonstrated a useful and practical method for planning an insecticide spraying area systematically. The flexible parameters, including the user-defined size of spraying area and the weight of the boundary minimization can be adjusted according to the amounts of resources and the experience of public health workers. In addition, a dengue epidemic will evolve over time. The utilization of the three epidemic indicators (i.e. frequency, duration and intensity) to identify the temporal and spatial clusters was demonstrated to be a suitable indicator in addition to the traditional case incidence data [11]. As a result, by including those indicators, the effectiveness at vector control and planning the appropriate area for spraying is enhanced.

The disease frequency, intensity and duration maps, however, clearly show that the main hotspot and several additional areas (e.g. in Annan District or South District) were severely affected by dengue fever in 2007. These regions are not covered by any of the optimized spraying areas (cases 1-6) in the model. If the weighting of minimal boundary in the objective function is decreased, these regions can be increasingly covered by the scattered hotspots. Thus, the model is able to aid in planning spraying when there are several scattered or clustered hotspots of dengue fever cases. Many tropical developing countries have been suffering the threat of the dengue fever and this model might help them plan the spraying area efficiently and economically. The approach can be used in any environments when the data is available. Before applying our model into practical use, there are some principal data you need to have such as dengue surveillance data (i.e. onset dates and locations of the dengue cases) and base GIS layers (i.e. administrative boundaries and road maps). In the model, the major parameters are only the weights of objective functions and the size of planning area.

The dengue control guidelines from Taiwan’s CDC [12] mention the standard operating procedure when a local department of health receives notification of suspected dengue infection cases from the hospitals or clinics. The public health workers need to do source reduction within 48 hours based on possible infection sites including living areas, work places and other possible infected places. Chemical space spraying will be applied when breeding sites are found in the outdoors. The strategy of space spraying is currently based on each individual case instead of a systematic preventive approach. With GIS application and our model, the compact or comprehensive spraying area would be planned based on the user’s experience during different stages of the epidemic. In addition, the temporal distribution of dengue infections has historically varied greatly in different months. Our model could be scaled down into daily or weekly units to immediately adjust to the variability of the epidemic.

Comparing our model’s planning area with the real spraying area, the major spraying areas for the dengue hotspots were consistent, retrospectively. Due to limitations on both data and feasibility of the study, we do not conduct a real experiment in ongoing epidemic control. Most studies for evaluating the effectiveness of peridomestic space spraying with insecticide used a quasi-randomized study design [13]. Therefore, it was easily confounded by other factors or breeding site reduction campaigns. Although the effectiveness of peridomestic space spraying in reducing dengue transmission has not been conclusively demonstrated, one empirical study in Taiwan showed that a significant drop in dengue incidence was correlated with an extensive emergency vector-control campaign in multiple space sprays [14]. In order to further validate our model in the future, the effectiveness evaluations in different space spraying strategies including current control policy and a GIS-aided model will be needed. In the practical integrated vector management (IVM) suggested by WHO [15], there are five key elements of an IVM strategy listed as follows.

1. Advocacy, social mobilization and legislation.

2. Collaboration within the health sector and with other sectors.

3. Integrated approach.

4. Evidence-based decision making.

5. Capacity-building.

The model proposed here mainly focuses on the third and fourth ones, especially aiding the spatial planning of the chemical vector control. Furthermore, our model also could be extended to routine vector surveillance when the epidemic indicators are replaced with entomological indicators such as the Breteau Index and House Index.

In the literature, the timing of using insecticide [16], the usage of the insecticide on the cover of water containers [17], and insecticide spraying on the breeding sites [18] were all shown to reduce dengue transmission. However, there is little known about how to arrange the spraying area when dengue cases occur. Hence, the core of the study used multi-objective planning to design an effective and systematic method for spraying area selection. Typically, multiple criteria simultaneously govern the hotspot selection process, such as the planning area, boundary length, and the coverage of epidemic risk. Reducing the boundary length of a hotspot area relative to its area is another critical concern in epidemic planning, owing to its influence on the economic costs of spraying.

## Conclusions

The proposed model in this study would allow the temporal variability of dengue based on incorporating a time-varying, predefined control area. An epidemic of dengue infections occurred with a distribution over both time and space. This study incorporated three critical indicators, namely frequency, duration and intensity of epidemic cases, into the multi-objective planning of insecticide spraying. Results showed that our planned spraying area was within the real, historical spraying area of the 2007 Tainan epidemic, but the planned area was more concentrated. The model provided an alternative way to obtain a cost-effective spraying area in controlling future dengue epidemics. In the future, enhancements to the model would incorporate exposure risk and entomological indicators, which would capture the dengue risk more effectively. The quasi-experiments on comparing effectiveness between traditional and GIS-aided space spraying were needed.

## Competing interests

The authors declare that we do not have any competing interests related to this study.

## Authors’ contributions

HJC designed the idea and formulation of the model and wrote the manuscript. TCC designed and guided the study and also revised the manuscript. FJJ did data processing and GIS analysis. All authors read and approved the final manuscript.
